# Selected Trichothecenes in Barley Malt and Beer from Poland and an Assessment of Dietary Risks Associated with their Consumption

**DOI:** 10.3390/toxins11120715

**Published:** 2019-12-09

**Authors:** Edyta Ksieniewicz-Woźniak, Marcin Bryła, Agnieszka Waśkiewicz, Tomoya Yoshinari, Krystyna Szymczyk

**Affiliations:** 1 Department of Food Analysis, Prof. Waclaw Dabrowski Institute of Agricultural and Food Biotechnology, Rakowiecka 36, 02-532 Warsaw, Poland; edyta.wozniak@ibprs.pl (E.K.-W.); krystyna.szymczyk@ibprs.pl (K.S.); 2 Department of Chemistry, Poznan University of Life Sciences, Wojska Polskiego 75, 60-625 Poznan, Poland; agnieszka.waskiewicz@up.poznan.pl; 3 Division of Microbiology, National Institute of Health Sciences, 3-25-26 Tonomachi, Kawasaki-ku, Kawasaki-shi, Kanagawa 210-9501, Japan; t-yoshinari@nihs.go.jp

**Keywords:** *Fusarium* toxins, modified mycotoxins, beer, malt, risk assessment

## Abstract

Eighty-seven samples of malt from several Polish malting plants and 157 beer samples from the beer available on the Polish market (in 2018) were tested for *Fusarium* mycotoxins (deoxynivalenol (DON), nivalenol (NIV)), and their modified forms ((deoxynivalenol-3-glucoside (DON-3G), nivalenol-3-glucoside (NIV-3G), 3-acetyldeoxynivalenol (3-AcDON)). DON and its metabolite, DON-3G, were found the most, among the samples analyzed; DON and DON-3G were present in 90% and 91% of malt samples, and in 97% and 99% of beer samples, respectively. NIV was found in 24% of malt samples and in 64% of beer samples, and NIV-3G was found in 48% of malt samples and 39% of beer samples. In the malt samples, the mean concentration of DON was 52.9 µg/kg (range: 5.3–347.6 µg/kg) and that of DON-3G was 74.1 µg/kg (range: 4.4–410.3 µg/kg). In the beer samples, the mean concentration of DON was 12.3 µg/L (range: 1.2–156.5 µg/L) and that of DON-3G was 7.1 µg/L (range: 0.6–58.4 µg/L). The concentrations of other tested mycotoxins in the samples of malt and beer were several times lower. The risk of exposure to the tested mycotoxins, following the consumption of beer in Poland, was assessed. The corresponding probable daily intakes (PDIs) remained a small fraction of the tolerable daily intake (TDI). However, in the improbable worst-case scenario, in which every beer bottle consumed would be contaminated with mycotoxins present at the highest level observed among the analyzed beer samples, the PDI would exceed the TDI for DON and its metabolite after the consumption of a single bottle (0.5 L) of beer.

## 1. Introduction

Barley (*Hordeum vulgare* L.) has been grown for many years and is of great economic importance [1]. Approximately 57 million tonnes of barley was produced annually (in 2018) in the European Union, while global production has reached 147 million tonnes annually [2]. Most of the harvested grain is used as feed but the highest quality barley is selected for food production, including the production of malt. Malt is an ample source of the B-group vitamins, niacin, and minerals. It is increasingly used in the bakery and pastry industries to improve the quality of both the taste and health of their products [3]. However, beer production remains as the main application of malt [1,4]. Beer is an alcoholic beverage commonly consumed in numerous countries globally. Poland has the third largest quantity of beer production in Europe (approximately 93, 40.5, and 40.4 million hectoliters in Germany, UK, and Poland, respectively) and the fourth highest beer consumption per capita in Europe (approximately 138, 105, 101, and 97 liters in Czech Republic, Austria, Germany, and Poland, respectively [5]).

To arrive at a high-quality malt, one needs to start with a healthy grain with sufficiently high energy for germination and sufficient protein content. However, unfavorable climatic conditions during the plant vegetation season may negatively impact the quality of the grain and consequently, the decrease quality of the malt produced from that grain [6]. The most important climatic conditions are rainfall and temperature, which are two factors that mostly determine the degree to which the plants may become infected with pathogen fungi. *Fusarium* is one of the major fungal species infecting cereal grains, including barley. *Fusarium* head blight (FHB) disease caused by these fungi is a problem in various regions of the world. The fungal infection decreases crop yield, but even greater damage may result from the production of mycotoxins, which are secondary metabolites of the fungi that are toxic to humans and animals [7].

*Fusarium* spp. most often responsible for FHB in Poland include *F. graminearum*, *F. avenaceum*, and *F. culmorum*; however, other species are also seen in various regions of the world [8,9,10]. The mycotoxins produced by *Fusarium* in cereal grains include the trichothecenes, deoxynivalenol (DON), and nivalenol (NIV), and their modified forms. These toxins are also phytotoxic [11,12]. *F. culmorum* and *F. graminearum* are among the varieties that most aggressively infect plant ears [13,14]. Many of these fungi are capable of synthesizing 3- (3-AcDON) or 15-acetyl deoxynivalenol (15-AcDON), which are modified forms of DON [15]. Studies of the phytotoxic effects of DON have shown that the ability to covert DON into deoxynivalenol-3-glucoside (DON-3G) is the plant’s primary defense mechanism against the toxin. Similar metabolic detoxication mechanisms help to build resistance to toxins in numerous cereal grain plants [16]. In barley, this mechanism is thought to be controlled by the QTL (quantitative trait loci)-specific region. Future studies involving deeper genetic analyses may help to develop tools to select fungal toxin-resistant plants using specific markers (marker-assisted selection; [17]). The phytotoxic effects of DON-3G are very weak compared to DON [18] and thus, it may be expected that a similar relationship holds for nivalenol 3-glucoside (NIV-3G) and NIV.

The consumption of DON- and/or NIV-contaminated food/feed may lead to disorders of the gastrointestinal tract, reproductive organs, and/or the immune system in both humans and animals. The toxicological characteristics of these toxins have been extensively described [19]. The lower levels of toxicity of DON-3G compared with DON have been confirmed in both humans and animals. In some in vitro studies and in some research on animals, it has been shown that DON-3G is not transported through the intestinal epithelium, but rather, is hydrolyzed by bacteria within the lower part of the alimentary tract [20]. Similar data are not available for NIV-3G, but it is commonly thought that the adverse effects of NIV-3G are weaker than those of NIV, as they are for DON-3G and DON.

Currently, the only European Commission regulation concerning mycotoxins in foodstuffs requires that the DON concentration in unprocessed cereal grains must not exceed 1250 µg/kg [21]. Taking into consideration the scientific evidence regarding the rapid absorption and excretion of DON, the in vivo deacetylation of 3- and 15-AcDON, and the hydrolysis of DON-3G in the lower parts of the alimentary tract; a European Food and Safety Authority (EFSA) expert panel recognized in 2017 that the toxic effects of DON-derivatives in humans may be comparable to the toxic effects of DON. Therefore, the tolerable daily intake (TDI) and reference dose (RfD) values have been recalculated as the sum of the three latter substances. Based on epidemiological data, a TDI threshold of 1 μg/kg body weight/day and an RfD dose of 8 μg/kg body weight/day have been accepted [19].

Reports on mycotoxins and their metabolites in Polish malts used in the brewing industry are very limited. The aims of this work included: (i) to assess the contamination of malts, sampled from several Polish malting plants, with selected *Fusarium* mycotoxins including their modified forms; (ii) to assess the mycotoxin contamination of beer available in 2019 on the Polish market; and (iii) to assess the risk of exposure to these mycotoxins following the consumption of beer in Poland.

## 2. Results and Discussion

### 2.1. Malt

Mycotoxins were found in the majority of the malt samples analyzed (Table 1). DON and DON-3G were found most often (in 90% and 91% of the malt samples, respectively) and at the highest levels (average of 52.9 and 74.1 µg/kg for DON and DON-3G, respectively). The percentage of samples positive for 3-AcDON was clearly lower 59% and NIV and NIV-3G were detected in the least number of samples (24% and 48%, respectively). DON-3G/DON molar ratios varied from 22% to 186% among DON-positive samples, while NIV-3G/NIV molar ratios varied from 32% to 126% among NIV-positive samples. Individual results regarding the content of individual mycotoxins in malt samples are presented in Table S1.

In grains, DON-3G is known to be a product of the plant defense reaction to the presence of the phytotoxin, DON [22,23,24]. DON-3G is easily soluble and plants can easily transport it from the cytoplasm to vacuoles or the intercellular space [16]. The DON-3G/DON ratio in the grain itself does not usually exceed 30% [25,26]. However, in malt samples we observed an average DON-3G/DON ratio of 89%, with a range of 22%–186%. Relatively high values (average 65%, range 32%–126%) were also noted for the NIV-3G/NIV ratio. Some researchers have suggested that changes occur during the malting process that activate secondary detoxicating enzymes, which then catalyze the conversion of the toxins to their glycoside derivatives [27,28,29]. Maul et al. [29] have shown that sprouting seeds of barley, millet, oat, rye, and spelt are capable of converting DON into DON-3G by means of UDP-glucosyltransferases. In barley, approximately 50% of DON was found to be converted, mainly into DON-3G, with a similar conversion rate observed in wheat. Moreover, Lancova et al. [28] reported that, during barley grain germination, the concentration of DON may decrease by 90%, while the concentration of DON-3G may markedly increase, to a level as high or several times higher than DON. Spanic et al. [30] presented data on mycotoxin levels in wheat varieties varying in Fusarium head blight resistance; the average content of DON-3G increased from 59.9 µg/kg in grain to 163.9 µg/kg in malt.

There are very few reports in the literature on the co-occurrence of DON/DON-3G and NIV/NIV-3G in brewing malts, even though such data are essential for regulating food safety. In the present study, we detected these substances in both malt and beer samples. However, the DON concentration did not exceed 750 µg/kg, the maximum permissible level in malt specified in EC Regulation 1881/2006, in any of the tested malt samples [21]. Practically, malt plants in Poland do not purchase grain contaminated with DON at levels above 1 mg/kg, while the maximum permissible level in grain is 1.25 mg/kg, as per EC Regulation 1881/2006 [21]. Mitteleuropäische Brautechnische Analyskomommision [31] recommends the inspection of each batch of grain offered to a malting plant for the presence of *F. graminearum* and *F. culmorum*. If mycelia are visible, they recommend the analysis of the grain for mycotoxins. There are some indications in the literature [32,33,34,35] that high amounts of additional mycotoxins may be synthesized in fungi-contaminated grain during the malting process, thus significantly impacting food safety.

### 2.2. Beer

The majority of beers marketed in Poland are light beers based on pilsner malts. However, dark ale or lager beers produced from Munich malts, usually obtained from lower quality grains [36], caramel malts or roasted pale ale malts are also popular. The two latter malts are enzymatically inactive; they are introduced in small amounts [37], to darken the beer and enhance its flavor. Wheat beers are also becoming increasingly common on the market. They are produced from barley malt, with the addition of at least 50% wheat or wheat malt. The flavor of these beers is unique, differing from the flavor of classical barley-only beers [38]. We divided our beer samples into three common categories for analysis: light, dark, and wheat beers. The percentage of mycotoxin-positive beer samples in all these groups was high (Table 2). Individual results regarding the content of mycotoxins in beer samples are presented in Table S1.

As was the case for malt samples, DON and DON-3G were the most frequently found toxins in beer samples, being present in 96% and 98% of light beer samples, respectively, and in all the samples of dark and wheat beers. Other mycotoxins, namely, 3-AcDON, NIV, and NIV-3G were found at lower levels in 69%, 25%, and 58%; 67%, 54%, and 63%; and 43%, 25%, and 42% of the light, dark, and wheat beer samples, respectively. The maximum DON (156.5 µg/L) and DON-3G (58.4 µg/L) concentrations were found in a light and a dark beer sample, respectively. The average levels of the three remaining tested mycotoxins ranged from 0.7 to 1.5 μg/L, i.e., they were approximately 6–20 times lower than the DON levels. The average DON-3G/DON and NIV-3G/NIV molar ratios ranged from 34% to 46% and 41% to 50%, respectively. Neither the mycotoxin concentrations nor their molar ratios were dependent on the beer category.

The alcohol content of beer depends on the extent to which the yeast ferments the sugars, which largely depends on the amount of grain and malt in the fermentation batch. Stronger beer requires more grain, which results in a higher risk of mycotoxin contamination [27,39,40]. Grain extracts used for beer production contain mainly sugars but may also contain dextrins, nitrogenous compounds (proteins), mineral salts, and other compounds, depending on the recipe used by the beer manufacturer [41]. Therefore, a comparison of the level of mycotoxin contamination in beers with different extract contents must be treated only as an approximation. Therefore, we re-organized the beer samples into three different categories: mild beers (0.5–5.0% alcohol, 3.5–12.5% extract), regular beers (5.1–6.0% alcohol, 6.8–16.0% extract), and strong beers (6.1–10.0% alcohol, 8.4%–21.0% extract; Table 3).

The number of positive samples and the concentration of the majority of the tested mycotoxins positively correlated with alcohol content in most cases. DON and DON-3G were the predominant toxins in 94% and 98% of mild beer samples, respectively, and in all samples of regular and strong beer, with average DON concentrations of 7.1, 12.1, and 17.3 μg/L and average DON-3G concentrations of 5.6, 7.0, and 8.6 μg/L for mild, regular, and strong beers, respectively. Less clear, but similar trends were noted for the other tested mycotoxins.

Mycotoxin contamination of beer has been studied by numerous groups (Table 4). However, data on the co-occurrence of DON, DON-3G, 3-AcDON, NIV, and NIV-3G in beer are scarce. The scope of most reported studies has been restricted to DON, DON-3G, and 3-AcDON, with a few studies also including NIV. Typically, the reported concentrations of the predominant DON have not exceeded 100 µg/L [27,42,43,44,45]. The findings from the present study mostly agree with those from previous studies (because the fraction of positive samples may depend on the LOD and LOQ of the method used). Higher concentrations of DON have been found mainly in beers originating from non-European countries, including craft beers from Brazil (127–501 µg/L; [46]), traditional African beers from Cameroon (140–730 µg/L; [47]), and Busaa-type beers from Kenya (200–360 µg/kg [48]). However, relatively high DON concentrations (104–182 μg/L) have also been found in strong (>8% alcohol) Norwegian Imperial Stout beer [49]. In this study, we found a high DON concentration (156.5 μg/L) only in one strong (>8% alcohol) sample of a light beer.

Some of the beer samples tested had a higher concentration of DON-3G than DON. Similar DON-3G/DON molar ratios have been reported in the literature, with averages of 0.56 (range 0.11–1.25 [43]) and 0.79 (range 0.1–2.6 [49] and 0.7–1.0 [26]. As can be seen, the DON-3G/DON molar ratios in beer are similar to those in malt.

### 2.3. Dietary Exposure Assessment

The following group TDI values were used in the assessment of risk of exposure to mycotoxins following beer consumption: 1 µg/kg body weight/day of the sum of DON, DON-3G, 3-AcDON, and 15-AcDON [20] and 1.2 µg/kg body weight/day of the sum of NIV and NIV-3G [50]. The average beer consumption in Poland is 97 L per capita annually, i.e., 0.27 L per capita per day [5]. In three considered scenarios, it was assumed that consumed beer contained mycotoxins at a level equal to: (i) the median, (ii) the third quartile, or (iii) the maximum concentration found in our samples (the worst-case scenario). It was assumed that the average adult in Poland weighs 70 kg. The results of the calculations are shown in Table 5. PDI values remained a small fraction of TDI values in the first and second scenarios (5.1% and 7.9%, respectively, for DON and its derivatives and 0.32% and 0.61%, respectively, for NIV and its derivatives). In the improbable third scenario (worst case), the PDI would reach 65.2% of the TDI for DON and its derivatives and 2.41% of the TDI for NIV and its derivatives.

The average consumption of 0.27 L of beer per day assumed in the above dietary exposure assessment does not reflect the real situation, since beer consumers rarely drink less than one bottle (0.5 L) per day. The PDI for persons drinking 0.5 L of beer daily would be approximately twice the values calculated above, in which case the TDI of DON and its derivatives would exceed the worst-case scenario by approximately 30%. Each additional beer bottle consumed per day would double the above calculated PDI values. It is also worth noting that the analytical method developed here was not efficient at detecting 15-AcDON. However, since 3-AcDON was detected at very low levels, one can expect that the contribution of 15-AcDON to the PDI is insignificant.

Of course, beer is not the main source of DON and its derivatives (the most important trichothecenes from a food safety point of view) in the human diet. Greater levels of exposure come from the consumption of bakery products, corn flakes, pasta, and other grain-based foodstuffs that are consumed daily, not only by beer consumers. Considering the exposition, bakery products and pastas are in Europe more and more often indicated as a possible quite serious threat to human health [50]. Studies of markers in urine have shown that chronic exposure to DON and its derivatives is greater than the accepted TDI [51,52,53]. Therefore, the consumption of beer may increase the risk of excessive mycotoxin exposure.

Data on the risks associated with the consumption of mycotoxin-contaminated beers exist only with respect to officially regulated toxins. It is a common observation that DON is the greatest risk factor, but beer is not generally considered an important source of dietary mycotoxin exposure. Even if the maximum detected DON concentrations are taken into account, the PDI values remain a small percentage of the TDI values, regardless of the country of origin of the beer. For example, the PDI is 14.0–20.8% of the TDI in Poland [54]; 18% of the TDI in Brazil [46]; 0.15–6.14% of the TDI in Spain, where the average consumption is just half of that in Poland [55]; 0% of the TDI in Cyprus and 10% of the TDI in Ireland [56].

The consumption of mycotoxin-contaminated beer results in negligible risk of exposure to NIV and NIV-3G. EFSA has reported that even the consumption of bakery products and pasta is safe in terms of exposure to these toxins [57]. In view of the low concentrations of NIV and NIV-3G, the PDI values are far below the TDI values, even for foodstuffs that are consumed in relatively large quantities, such as bakery products and pasta.

## 3. Conclusions

The data presented here on the co-occurrence of DON, NIV, and their metabolized (masked) forms in brewing malts and beers available on the Polish market are among the first reported in the literature. Mycotoxins were found in the majority of the barley malt and beer samples tested. DON and its metabolite, DON-3G, were found most frequently (in more than 90% of samples), although at safely low levels. NIV and its metabolite, NIV-3G, were found at lower levels in malt and beer samples. Because of the low mycotoxin levels, none of the tested beers were regarded as unsafe from a toxicological point of view. However, in the worst-case scenario, the PDI would exceed the TDI for DON and its metabolites after drinking just one bottle (0.5 L) of beer.

## 4. Materials and Methods

### 4.1. Reagents and Standards

Certified reference standards of DON, 3-AcDON, and NIV (100 μg/mL in acetonitrile), and DON-3G (50 μg/mL in acetonitrile:water, 50:50, *v*/*v*), were purchased from Romer Labs (Tulln, Austria). NIV-3G (110 μg/mL) was isolated from wheat, according to the procedure described by Yoshinari et al. [58]. Acetonitrile, methanol, and LC/MS-grade water were purchased from Witko (Łódź, Poland). Ammonium formate and formic acid (LC-MS grade) were obtained from Fisher Scientific (Millersburg, PA, USA). DON-NIV wide-bore (WB) immunoaffinity columns and PBS buffer solutions were purchased from Vicam (Watertown, NY, USA).

### 4.2. Research Material

One hundred and fifty-seven beer samples and 87 barley malt samples were analyzed. Various brands of light, dark, and wheat beers (mild, regular, and strong) were purchased in 2019 from local supermarkets in Poland. Malt was sampled from various malt plants located throughout the country, in line with the guidelines specified within EC Regulation 519/2014 (February 23, 2006) [59], which describes sampling and analysis methods for the official control of mycotoxin levels in foodstuffs. All the acquired samples belonged to the most common Pilsner malts, which are used to produce pale straw-colored ale and lager beers [36]. Malt samples, each with a mass of approximately 1 kg, were ground in a Knife Mill Grindomix GM 200 grinder (Retsch GmbH, Haan, Germany).

### 4.3. Sample Preparation

Malt and beer samples were prepared for analysis using a method previously described by our research team [42,60]. After extraction and homogenization (for malt extraction in Unidrive 1000 homogenizer, CAT Scientific Inc., Paso Robles, CA, USA), each sample was passed through a DON-NIV WB immunoaffinity column at a speed of 1–2 drops/s. The column was rinsed with 10 mL of PBS and 10 mL of de-ionized water. Analytes were washed out of the column, first with 0.5 mL of methanol and then with 1.5 mL of acetonitrile and were collected into a reaction vial. The solvent was evaporated in a stream of nitrogen. The residues were re-dissolved in 300 µL of 30% methanol and analyzed by liquid chromatography-mass spectrometry (LC-MS). Samples were analyzed at three replications.

### 4.4. LC-MS Analysis

An H-class liquid chromatograph coupled to a mass spectrometer with a time-of-flight analyzer (UPLC-TOF-HRMS; Waters, Milford, MA, USA) was used to analyze mycotoxins. Analytes were separated on a 2.1 × 100 mm, 1.6 µm UPLC C18 Cortecs chromatographic column (Waters) with an appropriate pre-column, operated with a gradient regime. Phase A was 90:10 *v*/*v* methanol:water, phase B was 10:90 *v*/*v* methanol:water. Both phases contained 0.2% formic acid and 10 mM ammonium formate. The flow rate was 0.3 mL/min, with the following flow gradient: 0–2 min, 100% B; 3–6 min, 50% B; 22–23 min, 100% A; and 25–28 min, 100% B. Five microliters of each sample was injected onto the column. The mass spectrometer was operated in the positive/negative electrospray ionization mode, with an ion source temperature of 150 °C and a desolvation temperature of 300/350 °C for positive/negative ionization, respectively. The nebulizing gas (N_2_) flow rate was 750 L/min and the cone gas flow rate was 40 L/min. The capillary bias was 3200 V. Ion optics was operated in V mode and the instrument was calibrated using a leucine-enkephalin solution.

### 4.5. Method Validation

Linearity ranges, limits of detection (LOD, the concentration at which the signal:noise ratio was 3), limits of quantification (LOQ, the concentration at which the signal:noise ratio was 10), recovery rates (R), and repeatability/precision (expressed as the relative standard deviation [RSD]), were determined using calibration curves that were constructed using separate blank samples for each mycotoxin of interest in the beer and malt matrices. The blanks were prepared in the same way as the analytes, except that the respective amount of standard mixture was added just prior to finally dissolving it in 30% methanol, after which the solvent was removed in a dry nitrogen stream. Each calibration curve consisted of eight points. The concentrations covered for the malt samples (in µg/kg) were: 5.0–1028 for DON; 4.0–516 for DON-3G; 2.0–1028 for 3-AcDON; 8.0–1050 for NIV; and 5.0–565 for NIV-3G. The concentrations covered for the beer samples (in µg/L) were: 3–68.6 for DON; 2.1–34.4 for DON-3G; 0.9–68.6 for 3-AcDON; 2.1–70.1 for NIV; and 1.6–37.7 for NIV-3G. The results of the analytical method validation experiment are shown in Table 6 and Table 7.

Since all analytes of interest belonged to the trichothecenes group, we assessed the performance of the method for DON analysis using the following specifications listed in EC Regulation 519/2014 [59]: recovery rates 60%–110% or 70%–120%, depending on the fortification level and RSD ≤20%. These criteria were met in 34 out of 35 analyte/fortification level combinations. In one case, the RSD was above 20%.

This validated method was then used to analyze DON, DON-3G, 3-AcDON, NIV, and NIV-3G in the malt and beer samples.

## Figures and Tables

**Table 1:** Concentration of mycotoxins in 87 barley malt samples.

Assumed Values	Concentration (μg/kg)					Molar Ratios	
	DON	DON-3G	3-AcDON	NIV	NIV-3G	DON-3G /DON	NIV-3G /NIV
Positive samples (%)	78 (90%)	79 (91%)	51 (59%)	21 (24%)	42 (48%)	78 (90%)	21 (24%)
Average	52.9	74.1	7.7	22.1	13.9	89%	65%
Median	24.2	33.1	4.9	17.5	10.0	88%	66%
Min–Max	5.3–347.6	4.4–410.3	2.2–40.2	8.3–118.6	5.0–57.4	22%–186%	32%–126%

DON, deoxynivalenol; DON-3G, deoxynivalenol-3-glucoside; 3-AcDON, 3-acetyldeoxynivalenol; NIV, nivalenol; NIV-3G, nivalenol-3-glucoside.

**Table 2:** Concentration of mycotoxins in light, dark, and wheat beers.

Type of Beer		Concentration (μg/L)					Molar Ratios	
		DON	DON-3G	3-AcDON	NIV	NIV-3G	DON-3G /DON	NIV-3G /NIV
Light beers (*n* = 105)	No. of positive samples (%)	101 (96%)	103 (98%)	72 (69%)	70 (67%)	45 (43%)	100 (95%)	42 (40%)
	Average	13.0	7.3	1.0	1.5	1.1	46%	42%
	Median	8.0	4.8	0.7	1.4	0.8	33%	30%
	Min–Max	1.2–156.5	0.6–36.8	0.3–8.3	0.6–3.6	0.5–4.5	10–149%	12–137%
Dark beers (*n* = 28)	No. of positive samples (%)	28 (100%)	28 (100%)	7 (25%)	15 (54%)	7 (25%)	28 (100%)	6 (21%)
	Average	11.7	7.8	1.2	1.0	0.7	40%	41%
	Median	8.8	4.8	0.8	0.8	0.6	39%	36%
	Min–Max	2.7–54.4	1.3–58.4	0.3–3.9	0.6–2.5	0.5–0.8	18–71%	30–74%
Wheat beers (*n* = 24)	No. of positive samples (%)	24 (100%)	24 (100%)	13 (58%)	15 (63%)	10 (42%)	24 (100%)	9 (38%)
	Average	9.6	5.0	0.9	1.1	0.9	34%	50%
	Median	9.9	3.8	0.9	1.0	0.9	34%	52%
	Min–Max	2.2–24.6	0.6–13.2	0.9–1.9	0.6–2.0	0.5–1.6	14–59%	23–79%
Total (*n* = 157)	No. of positive samples (%)	153 (97%)	155 (99%)	92 (59%)	100 (64%)	62 (39%)	152 (97%)	57 (36%)
	Average	12.3	7.1	1.0	1.3	1.1	43%	43%
	Median	8.6	4.8	0.8	1.2	0.8	38%	37%
	Min–Max	1.2–156.5	0.6–58.4	0.3–8.3	0.6–3.6	0.5–4.5	10–149%	12–137%

DON, deoxynivalenol; DON-3G, deoxynivalenol-3-glucoside; 3-AcDON, 3-acetyldeoxynivalenol; NIV, nivalenol; NIV-3G, nivalenol-3-glucoside.

**Table 3:** Concentrations of mycotoxins in mild, regular, and strong beers.

Type of Beer		Concentration (μg/L)					Molar Ratios	
		DON	DON-3G	3-AcDON	NIV	NIV-3G	DON-3G /DON	NIV-3G /NIV
Mild beers *n* = 48)	No. of positive samples (%)	45 (94%)	47 (98%)	26 (54%)	21 (44%)	18 (38%)	45 (94%)	15 (31%)
	Average	7.1	5.6	0.8	1.2	1.3	50%	58%
	Median	4.3	3.0	0.7	1.0	1.0	45%	43%
	Min–Max	1.4–24.6	0.6–30.9	0.3–2.7	0.6–2.3	0.5–4.5	18–149%	26–137%
Regular beers (*n* = 61)	No. of positive samples (%)	61 (100%)	61 (100%)	40 (67%)	45 (75%)	23 (38%)	59 (97%)	23 (38%)
	Average	12.1	7.0	0.9	1.5	1.1	42%	41%
	Median	9.5	5.2	0.8	1.3	0.9	37%	37%
	Min–Max	1.2–54.2	0.6–31.5	0.4–2.6	0.6–3.6	0.5–2.8	15–118%	16–90%
Strong beers (*n* = 48)	No. of positive samples (%)	48 (100%)	48 (100%)	27 (56%)	34 (71%)	21 (44%)	48 (100%)	19 (40%)
	Average	17.3	8.6	1.3	1.3	0.7	38%	34%
	Median	8.5	5.2	0.9	1.1	0.7	37%	32%
	Min–Max	2.0–156.5	0.6–58.4	0.3–8.3	0.6–3.3	0.5–1.8	10–104%	12–52%

DON, deoxynivalenol; DON-3G, deoxynivalenol-3-glucoside; 3-AcDON, 3-acetyldeoxynivalenol; NIV, nivalenol; NIV-3G, nivalenol-3-glucoside.

**Table 4:** Selected literature data on mycotoxins in beer.

Beer	No. of Samples	Toxin	LOD (µg/L)	LOQ (µg/L)	Concentration(µg/L)		Reference
					Average	Max	
Wheat beer	46	DON	1	4.5	18.4	49.6	[43]
		DON-3G	0.9	3.5	11.5	28.4	
		3-AcDON	2.2	8.2	<LOD	<LOD	
Pale beer	217	DON	2.2	5.4	12	89.3	
		DON-3G	0.4	3.5	9.3	81.3	
		3-AcDON	2.4	6.8	<LOD	<LOD	
Dark beer	47	DON	2.9	11	22.4	45	
		DON-3G	1.4	4.1	10.7	26.2	
		3-AcDON	4.3	11	<LOD	<LOD	
Bock beer	20	DON	1.2	4.1	13.8	27.1	
		DON-3G	0.5	1.5	14.8	33.3	
		3-AcDON	3.6	9.2	<LOD	<LOD	
Non-alcoholic beer	19	DON	1.2	3	14.8	33.3	
		DON-3G	0.4	1.4	3	6.6	
		3-AcDON	2.6	6	<LOD	<LOD	
Shandy beer	25	DON	1.5	3.9	6.9	12.7	
		DON-3G	0.4	1.3	3.8	7.9	
		3-AcDON	2.7	10	<LOD	<LOD	
Wheat beer	10	DON	1	4.5	14	27	[44]
		DON-3G	0.9	3.5	8.6	15	
		3-AcDON	2.2	8.2	<LOD	<LOD	
Pale beer	10	DON	2.2	5.4	13	30	
		DON-3G	0.4	3.5	8.3	19	
		3-AcDON	2.4	6.8	<LOD	<LOD	
Dark beer	10	DON	2.9	11	11	11	
		DON-3G	1.4	4.1	9.6	16	
		3-AcDON	4.3	11	<LOD	<LOD	
Bock beer	10	DON	1.2	4.1	13	22	[44]
		DON-3G	0.5	1.5	16	32	
		3-AcDON	3.6	9.2	<LOD	<LOD	
Non-alcoholic beer	10	DON	1.2	3	3.7	3.7	
		DON-3G	0.4	1.4	2.3	3.1	
		3-AcDON	2.6	6	<LOD	<LOD	
Shandy beer	10	DON	1.5	3.9	6.4	6.4	
		DON-3G	0.4	1.3	3.5	5.5	
		3-AcDON	2.7	10	<LOD	<LOD	
Light beers	158	DON	1	2.5	1.6–9.2 (depending on alcohol content)	3.7–35.9	[27]
		DON-3G	1	2.5	1.7–5.8	1.2–37	
		AcDONs	2	5	1.7–5.8	1.0–25	
		NIV	2.5	10	<LOD	<LOD	
Dark beers	18	DON	1	2.5	1.3–11.2	1.0–16.0	
		DON-3G	1	2.5	<LOQ–7.8	<LOQ–26.0	
		AcDONs	2	5	<LOQ–13.7	<LOQ–24.0	
		NIV	2.5	10	<LOD	<LOD	
African traditional beer	10	DON	n.r	10	81.8	140	[49]
		DON-3G		2.5	<LOD	<LOD	
		AcDONs		10	<LOQ	<LOQ	
		NIV		5	8.7	9	
Bock beer	2	DON	n.r.	10	52	64	
		DON-3G		2.5	60	97	
		AcDONs		10	<LOD	<LOD	
		NIV		5	<LOD	<LOD	
Dark lager	2	DON	n.r.	10	32.5	41	
		DON-3G		2.5	52	68	
		AcDONs		10	<LOD	<LOD	
		NIV		5	<LOD	<LOD	
Double India Pale Ale	1	DON	n.r.	10	67	67	
		DON-3G		2.5	48	48	
		AcDONs		10	<LOD	<LOD	
		NIV		5	<LOD	<LOD	
Eisbock	1	DON	n.r.	10	32	32	[49]
		DON-3G		2.5	32	32	
		AcDONs		10	<LOD	<LOD	
		NIV		5	<LOD	<LOD	
Fruit/Vegetable/Spice	1	DON	n.r.	10	<LOQ	<LOQ	
		DON-3G		2.5	LOD	LOD	
		AcDONs		10	<LOD	<LOD	
		NIV		5	<LOD	<LOD	
Imperial Stout	18	DON	n.r.	10	95.1	412	
		DON-3G		2.5	96.7	619	
		AcDONs		10	<LOD	<LOD	
		NIV		5	<LOD	<LOD	
India Pale Ale	3	DON	n.r.	10	40	64	
		DON-3G		2.5	14	18	
		AcDONs		10	<LOD	<LOD	
		NIV		5	<LOD	<LOD	
Non/Low Alcohol	1	DON	n.r.	10	<LOQ	<LOQ	
		DON-3G		2.5	<LOD	<LOD	
		AcDONs		10	<LOD	<LOD	
		NIV		5	<LOD	<LOD	
Pale ale	5	DON	n.r.	10	20.3	40	
		DON-3G		2.5	29.5	82	
		AcDONs		10	<LOQ	<LOQ	
		NIV		5	<LOD	<LOD	
Pale Lager	6	DON	n.r.	10	12.5	13	
		DON-3G		2.5	22	53	
		AcDONs		10	<LOQ	<LOQ	
		NIV		5	<LOD	<LOD	
Smoked	1	DON	n.r.	10	23	23	
		DON-3G		2.5	14	14	
		AcDONs		10	<LOQ	<LOD	
		NIV		5	<LOD	<LOD	
Sour Ale	4	DON	n.r.	10	17	29	[49]
		DON-3G		2.5	16.7	22	
		AcDONs		10	<LOD	<LOD	
		NIV		5	<LOD	<LOD	
Stout	4	DON	n.r.	10	28	30	
		DON-3G		2.5	41.3	52	
		AcDONs		10	<LOD	<LOD	
		NIV		5	<LOD	<LOD	
Strong Dark Pale	3	DON	n.r.	10	17.5	25	
		DON-3G		2.5	26.5	35	
		AcDONs		10	<LOQ	<LOD	
		NIV		5	<LOD	<LOD	
Strong Pale Ale	9	DON	n.r.	10	17.5	25	
		DON-3G		2.5	26.5	35	
		AcDONs		10	<LOQ	<LOD	
		NIV		5	<LOD	<LOD	
Strong Pale Lager	1	DON	n.r.	10	12	12	
		DON-3G		2.5	17	17	
		AcDONs		10	<LOQ	<LOD	
		NIV		5	<LOD	<LOD	
Wheat beer	5	DON	n.r.	10	10	32	
		DON-3G		2.5	4	41	
		AcDONs		10	<LOQ	<LOD	
		NIV		5	<LOD	<LOD	
Mild beer	28	DON	1.3	4.1	10.5	65	[42]
		DON-3G	1.9	6.2	7.6	25	
		NIV	0.6	2.1	2.7	4.8	
Regular beer	34	DON	1.3	4.1	6.6	19.7	
		DON-3G	1.9	6.2	8.8	35.8	
		NIV	0.6	2.1	1.5	7.4	
Strong beer	38	DON	1.3	4.1	10	73.6	
		DON-3G	1.9	6.2	10.3	35.2	
		NIV	0.6	2.1	2.8	7.6	

n.r. = not reported.

**Table 5:** Group probable daily intake and its share of the total daily intake calculated in three scenarios, in which different concentrations of mycotoxins were assumed in the consumed beer.

Assumed Values	DON			DON+DON3G+3AcDON			NIV+NIV3G		
	Concentration (µg/L)	* PDI (ng/kg b.w./day)	%TDI	Concentration (µg/L)	PDI (ng/kg b.w./day)	%TDI	Concentration (µg/L)	PDI (ng/kg b.w./day)	%TDI
Median **	8.3	31.5	3.2	14.2	50.7	5.1	1.1	3.8	0.32
Quartile 3 **	13.3	50.4	5.0	22.2	79.4	7.9	2.1	7.3	0.61
Maximum	156.5	594.1	59.4	182.5	651.8	65.2	8.1	28.9	2.41

DON, deoxynivalenol; DON-3G, deoxynivalenol-3-glucoside; 3-AcDON, 3-acetyldeoxynivalenol; NIV, nivalenol; NIV-3G, nivalenol-3-glucoside; PDI, probable daily intake; TDI, total daily intake; * PDI = $\frac{C*C_{d}}{b.w.}$ , where C is concentration of the mycotoxin in the contaminated beer, C_d_ is the average daily consumption of beer in Poland, and b.w. is mean body weight. ** If the measurement for any analyte was below the LOQ, the median and 3rd quartile were calculated assuming that the analyte was present at the level of LOQ/2.

**Table 6:** Limits of detection, limits of quantification, and determination coefficients for individual analytes determined in malt and beer samples.

Analyte	Ion Mass (*m*/*z*)	Retention Time (min)	Malt			Beer		
			LOD (μg/kg)	LOQ (μg/kg)	*R^2^*	LOD (μg/L)	LOQ (μg/L)	*R^2^*
DON	341.2 (M+FA−H)^−^	4.08	5	17	0.9891	0.6	2.1	0.9977
DON-3G	503.2 (M+FA−H)^−^	4.22	4	13	0.9910	0.5	1.6	0.9919
3-AcDON	339.2 (M+H)^+^	4.98	2	7	0.9974	0.3	0.9	0.9899
NIV	357.2 (M+FA−H)^−^	2.38	8	24	0.9909	1.0	3.0	0.9889
NIV-3G	519.2 (M+FA−H)^−^	2.45	5	17	0.9905	0.6	2.1	0.9989

DON, deoxynivalenol; DON-3G, deoxynivalenol-3-glucoside; 3-AcDON, 3-acetyldeoxynivalenol; NIV, nivalenol; NIV-3G, nivalenol-3-glucoside; LOD, limit of detection; LOQ, limit of quantification; *R^2^*, determination coefficient.

**Table 7:** Recovery rates and relative standard deviations for individual analytes determined in malt and in beer samples spiked at different fortification levels.

Analyte	Malt (*n* = 4)			Beer (*n* = 4)		
	Fortification Level (μg/kg)	R (%)	RSD (%)	Fortification Level (μg/L)	R (%)	RSD (%)
DON	42.9 128.6 514.3 1028.5	90.7 94.3 101.3 97.0	12.9 8.2 11.9 15.0	17.1 34.3 68.6	75.0 106.0 85.0	8.8 2.8 9.5
DON-3G	21.5 64.5 258.1 516.2	87.4 73.5 89.9 79.1	6.1 9.8 11.1 15.4	8.6 17.2 34.4	87.0 93.0 89.0	6.7 2.5 6.0
3-AcDON	42.9 128.6 514.3 1028.5	105.1 105.4 103.8 102.1	18.4 4.7 8.8 22.1	17.1 34.3 68.6	93.0 97.0 87.0	6.7 2.7 6.7
NIV	43.8 131.4 525.6 1051.2	89.9 85.8 85.1 83.7	11.4 8.6 9.3 13.0	17.5 35.0 70.1	80.0 100.0 91.0	6.2 6.5 9.4
NIV-3G	23.6 70.7 282.8 565.6	105.0 85.0 87.7 86.4	13.7 7.8 9.6 13.1	9.4 18.9 37.7	93.0 101.0 96.0	6.7 6.6 8.0

DON, deoxynivalenol; DON-3G, deoxynivalenol-3-glucoside; 3-AcDON, 3-acetyldeoxynivalenol; NIV, nivalenol; NIV-3G, nivalenol-3-glucoside; R, recovery rate; RSD, relative standard deviation.

## Supplementary Materials

The following are available online at https://www.mdpi.com/2072-6651/11/12/715/s1, Table S1: Individual results of mycotoxin concentrations in the analyzed beer and malt samples.
